# Sketch of the Comparative Anatomy of the Nervous System; with Remarks on Its Development in the Human Embryo

**Published:** 1837-04

**Authors:** 


Art. XV.—*Sketch of the Comparative Anatomy of the Nervous System; with Remarks on its Development in the Human Embryo. With Plates.* By John Anderson, m.e.s., &c.—4to. pp. 63. *London*, 1837.

This is an useful compendium of known facts, collected and applied to illustrate the development of the human brain. The greater part of the work, as stated in the preface, has already appeared in the Medical Gazette of last year, but the author has done well in collecting the whole into a volume. It is justly remarked, also, that a work of this kind did not previously exist in our language. The plan which has been followed by the author has been to treat of the nervous system of different classes of animals, beginning with the lowest forms, and gradually ascending through the scale, comparing, as he passes along, the several different permanent conditions of the nervous system with the analogous transitory conditions of the human fœtal brain. In doing this, the author has rendered some service to comparative anatomy in this country; since, although we can discover but few new facts, those which are well known are placed in a favorable view to be understood. The work, however, gives evidence of being compiled by a young author, who has been somewhat too anxious to direct the attention of his readers to his own labours; and, in doing this, he has, unfortunately, sometimes forgotten to notice that he was only repeating the observations of others. There is also a little too much disposition to place his own observations and opinions,—which are often shown, by a few occasional errors, which have crept into them, to be from the hand of a young anatomist,—in opposition to those of the learned authorities whom he sometimes quotes. These are serious faults; because, in doing this, the author is not merely putting forward his own labours, to the exclusion of others, but he is in reality occasioning an injury to himself, by inducing those who would have gladly passed over minor errors, in consideration of his zeal and industry, to pause at such indications of egotism and vanity. Perhaps, when the opinions of the writer are a little more matured by experience, he will find it necessary to correct some of the statements in a second edition of the work; as, for instance, when he says that the lepidoptera have the most highly developed nervous system of all insects; an observation, the correctness of which admits of much doubt. Upon the whole, however, we are pleased with this work, which gives promise of something better than itself, and is highly creditable to the perseverance and industry of its author.

